# Comparative fitness of West Nile virus isolated during California epidemics

**DOI:** 10.1371/journal.pntd.0007135

**Published:** 2019-02-04

**Authors:** Gabriella Worwa, Andra A. Hutton, Aaron C. Brault, William K. Reisen

**Affiliations:** 1 Center for Vectorborne Diseases, Department of Pathology, Microbiology and Immunology, School of Veterinary Medicine, University of California, Davis, California, United States of America; 2 Division of Vector-Borne Diseases, Centers for Disease Control and Prevention, Fort Collins, Colorado, United States of America; Institute for Disease Modeling, UNITED STATES

## Abstract

West Nile virus (WNV) has been circulating in California since its first detection in 2003, causing repeated outbreaks affecting public, wildlife and veterinary health. Epidemics of WNV are difficult to predict due to the multitude of factors influencing transmission dynamics among avian and mosquito hosts. Typically, high levels of WNV amplification are required for outbreaks to occur, and therefore associated viral strains may exhibit enhanced virulence and mortality in competent bird species resulting in increased mosquito infection prevalence. In our previous study, most WNV isolates made from California during 2007–08 showed increased fitness when competed in House Finches (HOFI, *Haemorhous mexicanus*) and *Culex tarsalis* Coquillett mosquitoes against COAV997-5nt, a genetically marked recombinant virus derived from a 2003 California strain. Herein, we evaluated the competitive fitness of WNV strains isolated during California epidemics in 2004, 2005, 2007, 2011 and 2012 against COAV997-5nt. These outbreak isolates did not produce elevated mortality in HOFIs, but replicated more efficiently than did COAV997-5nt based on quantification of WNV RNA copies in sera, thereby demonstrating increased competitive fitness. Oral co-infections in *Cx*. *tarsalis* resulted in similar virus-specific infection and transmission rates, indicating that outbreak isolates did not have a fitness advantage over COAV997-5nt. Collectively, WNV isolates from outbreaks demonstrated relatively greater avian, but not vector, replicative fitness compared to COAV997-5nt, similar to previously characterized non-outbreak isolates of WNV. Our results indicated that ecological rather than viral factors may facilitate WNV amplification to outbreak levels, but monitoring viral phenotypes through competitive fitness studies may provide insight into altered replication and transmission potential among emerging WNV strains.

## Author summary

West Nile virus (WNV) is the cause of the largest mosquito-borne epidemic in the United States in recent history, with over 46,000 reported human cases to date, including >2000 deaths. Since the detection of WNV in California in 2003, repeated outbreaks have been associated with explosive WNV amplification in local mosquito and bird populations. Along with climatic and ecological factors, vector-host-pathogen interactions are potential drivers of WNV epidemics and warrant study. Here, we evaluated the competitive fitness of five WNV isolates made during Californian outbreaks from 2004–2012 by co-infection of *Culex tarsalis* mosquitoes and House Finches against a 2003 founding virus strain. Results indicated that competitive fitness in HOFIs was greater for four of the outbreak isolates, but coincidentally elevated mortality was not observed and viral RNA loads were lower compared to previously studied enzootic strains of WNV. Mosquito competitions resulted in levels of infection and transmission that did not differ among strains.

## Introduction

West Nile virus (WNV; *Flavivirus*, *Flaviviridae)* naturally cycles among competent avian hosts and *Culex* mosquitoes that serve as both amplification and bridge vectors [1]. Outbreaks of WNV in North America generally have occurred during the warm summer and fall months, when transmission spills over to include humans and other susceptible mammals [1]. Since its invasion of California in 2003 [2], WNV has not only adapted to and persisted in diverse habitats [3], but also has caused several epidemics, impacting public, veterinary and wildlife health [4–6]. Most regions of California now have experienced either year-round or repeated seasonal WNV activity [3]. Epidemics have been associated with intense WNV amplification and historically have followed a three-year pattern of introduction, rapid amplification and then subsidence [7]. Factors linked to WNV outbreaks have included above average temperatures and drought conditions, leading to enhanced virus transmission [8, 9]. Warming temperatures increase vector blood feeding frequency and shorten the extrinsic incubation period of the virus and in California are linked with reduced rainfall that results in increased domestic irrigation and mosquito production from urban drainage systems.

Rapid amplification also may relate to phenotypic differences between enzootic and epidemic isolates of WNV that enhance avian host and vector infection, facilitating transmission[10]. High titer avian viremias, associated with elevated mortality in certain avian hosts, are required to produce efficient mosquito infection [10] and have been a hallmark of WNV epidemics in North America [11]. Avian virulence in American crows has been related to single nucleotide polymorphisms in the NS3 and NS1-2B proteins [12–14]. After the 2004 WNV epidemic in Los Angeles, elevated passerine seroprevalence and corvid de-population were associated with limited WNV transmission during successive years [4]. However, the progressive loss of passerine flock immunity and corvid population recovery led to WNV resurgence in 2008 and again in 2011 [4]. Alternatively, decades of pathogen-host co-circulation may have led to a trade-off between a reduction in avian susceptibility as previously shown for House Sparrows (HOSPs; *Passer domesticus*) and increased WNV virulence leading to stable host competence [15]. Interestingly, the competence of several *Culex spp*. populations from California for the NY99 strain of WNV did not change significantly during WNV outbreak years compared to non-outbreak years [16], suggesting that epidemics were not linked to changes in vector competence.

Adaptive changes in arboviruses typically appear to be driven by viral diversification within mosquitoes [17], with subsequent expectoration of unique virus populations [18] followed by purifying selection in vertebrate hosts [19]. Studies with Venezuelan equine encephalitis virus (VEEV), for example, have shown that epizootic strains of VEEV originated from enzootic strains, but possessed less than 2% genetic diversity encoding for increased replicative phenotypes [20]. Enzootic isolates of WNV collected between 2007–08 from different regions of California possessed less than 0.2% genetic diversity and demonstrated increased replicative fitness compared to an isolate made during the initial introduction of WNV into California in 2003 [21]. Included in this study was a spring isolate from 2008 preceding the epidemic in Los Angeles that exhibited elevated fitness and virulence similar to the NY99 phenotype [22]. As a follow-up to these findings, five WNV isolates made during epidemics from 2004–2012 were assessed herein to determine if increased replicative fitness may facilitate amplification and thus be predictive of future outbreaks.

## Methods

### WNV isolates from California epidemics

Isolates of WNV were made from mosquito pool homogenates collected as part of the California Mosquito-Borne Encephalitis Virus Surveillance Program that had tested positive for WNV RNA by qRT-PCR [23]. One homogenate each was selected from outbreak years 2004, 2005, 2007, 2011 and 2012 from regions of California with high transmission activity (Table 1) and increased incidence of human cases. Supernatant from the selected mosquito pool homogenates was propagated once in African Green Monkey kidney cells (Vero cells; ATCC no. CCL-81) to generate sufficient material for all competition studies. The location, collection date, mosquito species, Ct value of the original mosquito pool homogenate, and infectious titer following Vero cell culture amplification were summarized in Table 1.

**Table 1:** Mosquito pools containing WNV collected during 2004–2012 California epidemics.

Isolate	Region	Collection date	*Culex* species	Ct	Titer ^a^
GRLA-04-1624	Encino, Los Angeles County	7/28/2004	*quinquefasciatus*	18.0	8.9±0.03
SAYO-05-912	Citrus Heights, Sacramento County	8/19/2005	*pipiens*	19.8	9.0±0.04
KERN-07-291	Bakersfield, Kern County	7/27/2007	*quinquefasciatus*	23.8	8.9±0.01
GRLA-11-6246	Los Angeles, Los Angeles County	9/02/2011	*quinquefasciatus*	19.2	9.3±0.02
SAYO-12-772	Sacramento, Sacramento County	5/23/2012	*tarsalis*	18.5	9.0±0.11

^a^ Mean±SEM infectious WNV titer of replicate Vero cell culture supernatants indicated as log_10_ plaque forming units (PFU) per mL.

The genetically labeled COAV997-5nt virus was utilized as a reference strain in competitions against all the wild type WNV isolates, because its fitness previously has been well characterized *in vitro* and *in vivo* [24, 25]. COAV997-5nt was generated from a clone of the COAV997 isolate collected in July 2003 from Imperial Valley, California, early in the WNV invasion of southeast California. Replicative fitness was assessed through competition in dually infected HOFI and *Cx*. *tarsalis* using 1:1 mixtures of equal titers of outbreak isolates and COAV997-5nt.

### Ethics in animal research statement

Depredation permits allowed trapping of wild HOFIs at vineyards near Bakersfield, CA under US Federal Fish and Game permit MB-082812-1 and State Fish and Game collecting permit SC-002281. *Culex* mosquitoes from which the WNV isolates were made were collected with dry-ice baited traps as part of the California Mosquito-Borne Encephalitis Virus Surveillance Program. Blood collection from chickens was approved under Institutional Animal Care and Use Committee (IACUC) protocol 15892 and permitted by the Kern Mosquito and Vector Control District in Bakersfield. Experiments with infectious WNV were performed in an animal biosafety level 3 facility containing aviaries and an insectary approved under USDA permit 47901 and Biological Use Authorization 0872 by the Environmental Health and Safety Institutional Committee of UC Davis. Animal experiments were conducted under protocol number 15895 approved by the UC Davis IACUC and under strict adherence to the American Veterinary Medical Association (AVMA) guidelines on the Care and Use of Laboratory Animals. UC Davis is approved for the use of animals in research under the National of Institutes of Health (NIH) assurance number A3433.

### In vivo fitness competitions

Infection, sampling and qRT-PCR assay methods essentially followed Worwa et al. [21, 24, 25]. Outbreak and reference viruses were competed within dually infected hosts, with wild type and the labelled COAV997-5nt clone detected by concurrent qRT-PCR assays. Briefly, equal concentrations of plaque forming units (PFU) of COAV997-5nt (8.34 log_10_ PFU per mL stock) and each outbreak isolate (stock titers in Table 1) were utilized to inoculate HOFIs and *Cx*. *tarsalis*. Mixed inocula were analyzed by qRT-PCR and differences in RNA numbers from COAV997-5nt and outbreak isolates expressed as ratios (outbreak isolate RNA divided by COAV997-5nt RNA) in Tables 2 and 3. To account for these relative RNA differences, the COAV997-5nt RNA copy data were normalized by multiplication with the RNA ratio determined for each inoculum.

**Table 2:** Blood meal exposure of *Culex tarsalis* females and resulting infection rates.

Isolate	*Cx*. *tarsalis* titer ^a^	RNA ratio ^b^	*Cx*. *tarsalis* (n) ^c^	Infection rate (%)^d^	Transmission rate (%)^e^
GRLA-04-1624	3.6±0.22	0.29	49	20	67
SAYO-05-912	4.1±0.07	0.02	71	21	67
KERN-07-291	3.8±0.12	0.47	88	43	73
GRLA-11-6246	4.0±0.09	0.10	88	31	71
SAYO-12-772	3.4±0.24	0.19	66	38	44

^a^ Mean±SEM titer of WNV (log_10_ PFU per mosquito) determined by Vero cell plaque assay in five fully engorged females per group collected immediately after blood meal exposure.

^b^ RNA ratios were calculated by dividing the mean RNA number of the outbreak isolate by COAV997-5nt detected in five fully engorged females of each group collected immediately after blood feeding and were used for normalization of competition samples collected on 14 dpi.

^c^ Number of surviving females collected on 14 dpi and tested for infection by RT-PCR. Total (n) equals 32 females from which expectorant was collected plus additional females that were frozen without expectorant collection.

^d^ Percentage of bodies that tested positive for WNV RNA by qRT-PCR for either the outbreak isolate, COAV997-5nt or both out of all surviving mosquitoes (n) per group.

^e^ Percentage of expectorants that tested positive for WNV RNA by qRT-PCR for either the outbreak isolate, COAV997-5nt or both out of WNV-positive bodies from 32 females from which expectorant was collected.

**Table 3:** HOFI competition challenge inocula.

Isolate	HOFI (n)	Challenge dose (PFU) ^a^	RNA ratio ^b^
GRLA-04-1624	6	101±4.5	0.04
SAYO-05-912	6	93±7.0	0.98
KERN-07-291	6	128±50	0.16
GRLA-11-6246	6	175±9.5	0.29
SAYO-12-772	6	175±27	0.20

^a^ Mean±SEM PFU of WNV in 50 μl of inocula administered to HOFIs as determined by plaque assay titration.

^b^ RNA ratios were calculated by dividing the mean RNA numbers from outbreak isolates by COAV997-5nt as detected in inocula and were used for normalization of competition samples collected between 1 and 7 dpi.

Fitness competitions in mosquitoes were performed using the KNWR laboratory-adapted colony of *Cx*. *tarsalis*, established in 2002 from collections made at the Kern National Wildlife Refuge, Kern County, California, which were reared in an insectary at 22°C with a photoperiod of 16:8 (L:D) hours. Females were transferred to a BSL-3 insectary and sugar starved for 24 hours prior to exposure to an infectious blood meal containing 7 log_10_ PFU per mL of each competition group. Blood meals were composed of heparinized WNV seronegative chicken blood spiked with 8 log_10_ PFU per mL of a 1:1 mix of outbreak isolates and COAV997-5nt, thereby diluting the virus mixture 10-fold in the blood meal. Sugar starved females were allowed to feed for 1 hour in the dark on pre-warmed blood meals offered through a Hemotek membrane system (Discovery Workshops, Accrington, Lancashire, UK). Five fully engorged females were frozen at -80°C immediately following blood meal exposure and were used for infectious blood meal analysis. The remaining blood-fed females were maintained at 26°C, 12:12 (L:D) and 60% humidity and daily offered a 10% sugar solution on cotton pads. After 14 days, all surviving females were anesthetized using triethylamine (Fisher Scientific, USA) to collect expectorants and then bodies as described previously [21, 25]. Expectorants were collected from 32 females per group using the capillary tube method. The remaining females were immediately frozen at -80°C and used to enhance sample sizes to estimate viral infection and numbers of RNA copies of each virus.

Wild, hatch-year HOFI were captured during the summer of 2012 using grain-baited traps at vineyards near Bakersfield, CA, transported to the Arbovirus Field Station at Bakersfield, treated with 0.2 mg per mL of chlortetracycline (Fort Dodge, Overland Park, KS) for two weeks, and tested for the presence of antibodies against WNV, St. Louis encephalitis (SLEV) and western equine encephalitis virus (WEEV) [26]. Seronegative birds were transported to UC Davis. After a 2-week acclimation period in the BSL-3 aviary, groups of six HOFIs were inoculated subcutaneously with 0.05 mL containing approximately 1,000 PFU of an equal mixture of COAV997-5nt and one of the outbreak isolates. Following challenge, HOFIs were evaluated for the presence of clinical signs until 14 days post infection (dpi), with 0.1 mL of blood collected daily by jugular venipuncture between 1–7 dpi and upon termination of the study at 14 dpi. HOFIs were euthanized by placement in a CO_2_ chamber when they were found moribund or at termination of the study on 14 dpi.

Mosquito bodies were homogenized twice for two minutes at 24 Hz using a Tissue Lyser (Qiagen, USA) and the suspension clarified by centrifugation at 5,000 × g and 4°C for 10 minutes. *Culex tarsalis* expectorants were amplified by one passage in Vero cell culture to enhance detection of low amounts of viral RNA. Nucleic acids were extracted from HOFI sera, mosquito homogenates and amplified expectorants using a MagMAX-96 Viral RNA isolation Kit (Applied Biosystems, USA). A previously described qRT-PCR assay was utilized for specific detection and quantification of viral RNA in mixed competition samples based on five nucleotide polymorphisms in the envelope gene region of COAV997-5nt [24]. Outbreak isolates were detected using primers previously described [27]. Infectious titers in mosquito blood meals and HOFI inocula were determined using plaque assay titration on Vero cells [12]. GraphPad Prism version 7 (La Jolla, CA) was used for statistical analysis and data plotting.

## Results

### *Culex tarsalis* fitness competitions

Concentrations of outbreak isolates and COAV997-5nt were measured in homogenates of five individual fully engorged females collected immediately following blood meal exposure of each group of *Cx*. *tarsalis* (Table 2). The number of WNV RNA copies present in each blood meal was expressed as the ratio of the outbreak isolate to COAV997-5nt (Table 2), and this was utilized for normalizing competition data [24]. Bodies and expectorants from surviving *Cx*. *tarsalis* were collected on 14 dpi and infection rates (Table 2) determined based on the percentage of bodies containing WNV RNA (outbreak isolate and/or COAV997-5nt).

**Fig 1:**
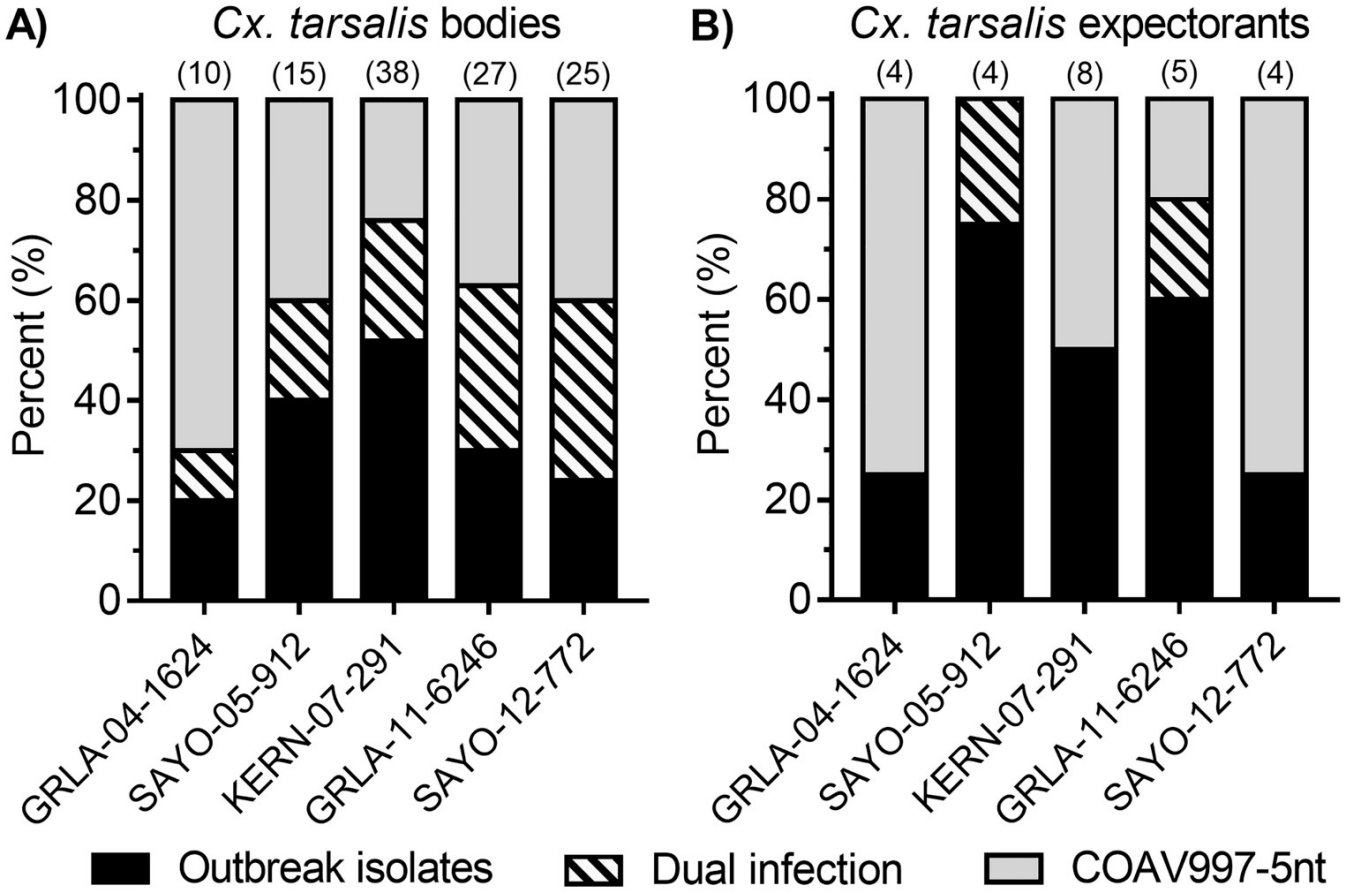
Percentage of *Culex tarsalis* bodies (A) and expectorants (B) positive for each outbreak isolate and/or COAV997-5nt viral RNA following competition. *Cx*. *tarsalis* bodies and cell-culture amplified expectorants were analyzed by concurrent qRT-PCR for the presence of viral RNA. Columns indicate the percentage of infected mosquitoes containing RNA for either the outbreak isolate (black color), COAV997-5nt (grey color) or both virus strains (grey with black stripes) within each competition group. The number of samples positive within each competition group was included in parentheses at the top of each column.

For each competition group containing one outbreak isolate and COAV997-5nt, we determined the percentage of *Cx*. *tarsalis* females with dually and singly infected bodies (Fig 1A) and cell culture amplified expectorants (Fig 1B).

Using a binominal proportions test, we compared expected equal proportions of singly/dually infected *Cx*. *tarsalis* and their expectorates to observed proportions specific for either the outbreak isolate or COAV997-5nt. Although dually infected bodies were present in all competition groups, GRLA-04-1624, KERN-07-291 and SAYO-05-912 groups contained greater numbers of singly infected *Cx*. *tarsalis* (P = 0.05); all expectorants following competition with isolates GRLA-04-1624, KERN-07-291 and SAYO-12-772 contained a single virus strain (P<0.001). The proportion of females infected with the outbreak isolates were not significantly different (P>0.05) compared to COAV997-5nt (Fig 1A) in all competition groups combined. Similarly, there was no difference (P>0.05) in the percentage of outbreak isolates and COAV997-5nt detected in the cell-culture amplified expectorants (Fig 1B), and equal numbers of singly infected expectorants contained either KERN-07-291 or COAV997-5nt in that group.

**Fig 2:**
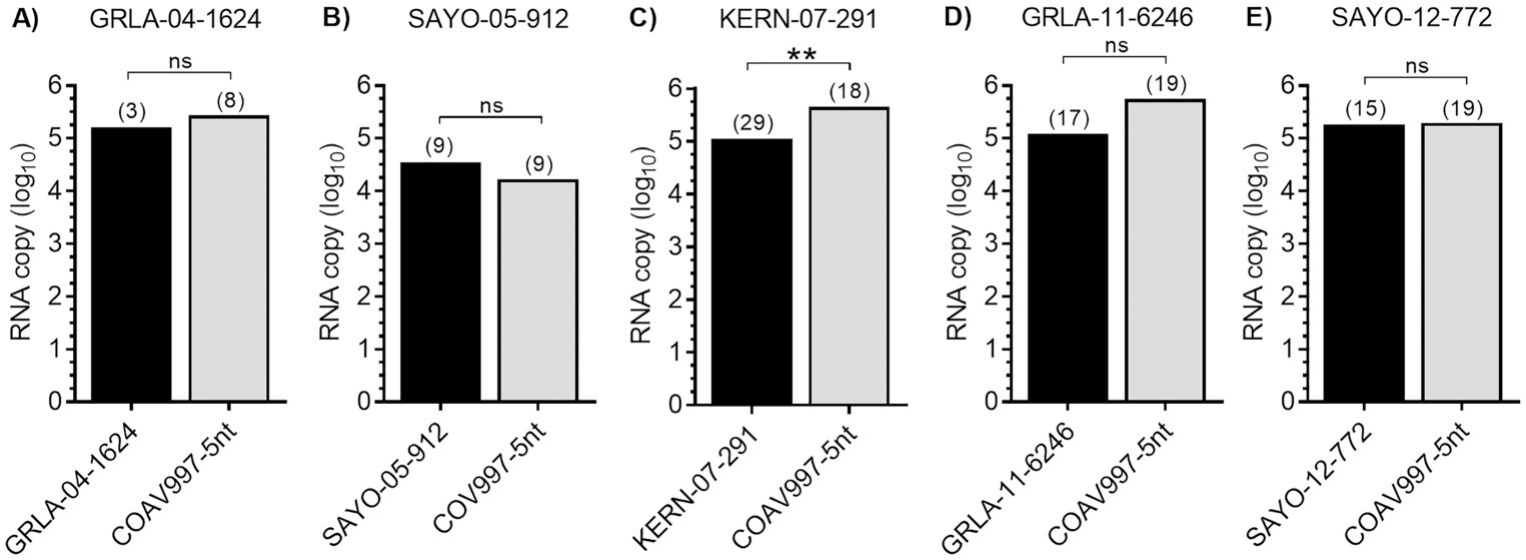
RNA copy numbers in bodies of infected *Cx*. *tarsalis*. Panels A to E show the average number of RNA copies within each competition group for outbreak isolates (black bars) and COAV997-5nt (grey bars) quantified by qRT-PCR in the bodies of singly and dually infected mosquitoes at 14 dpi. Number of samples is shown at the top of each column in parentheses. Statistical significance is indicated as “ns” for P>0.05 and double asterisk for P≤0.01 as determined by Mann-Whitney test.

There was no significant difference in the amount of RNA (transformed by y = log_10_) detected in *Cx*. *tarsalis* infected bodies following competition, except for isolate KERN-07-291 which replicated less efficiently (P = 0.006) in co-infected bodies compared to COAV997-5nt (Fig 2C). However, despite replicating to higher RNA copies in *Cx*. *tarsalis* bodies, COAV997-5nt was not transmitted at a significantly higher rate (P>0.05) compared to isolate KERN-07-291 (Fig 1B).

### HOFI fitness competitions

Each of the five outbreak isolates was competed against COAV997-5nt by dual inoculation of six seronegative HOFIs per competition group. The infectious titer and RNA concentration of the challenge inocula were determined by plaque assay titration and qRT-PCR, respectively, and RNA ratios determined for each group were used for normalization of mixed competition samples (Table 3).

**Fig 3:**
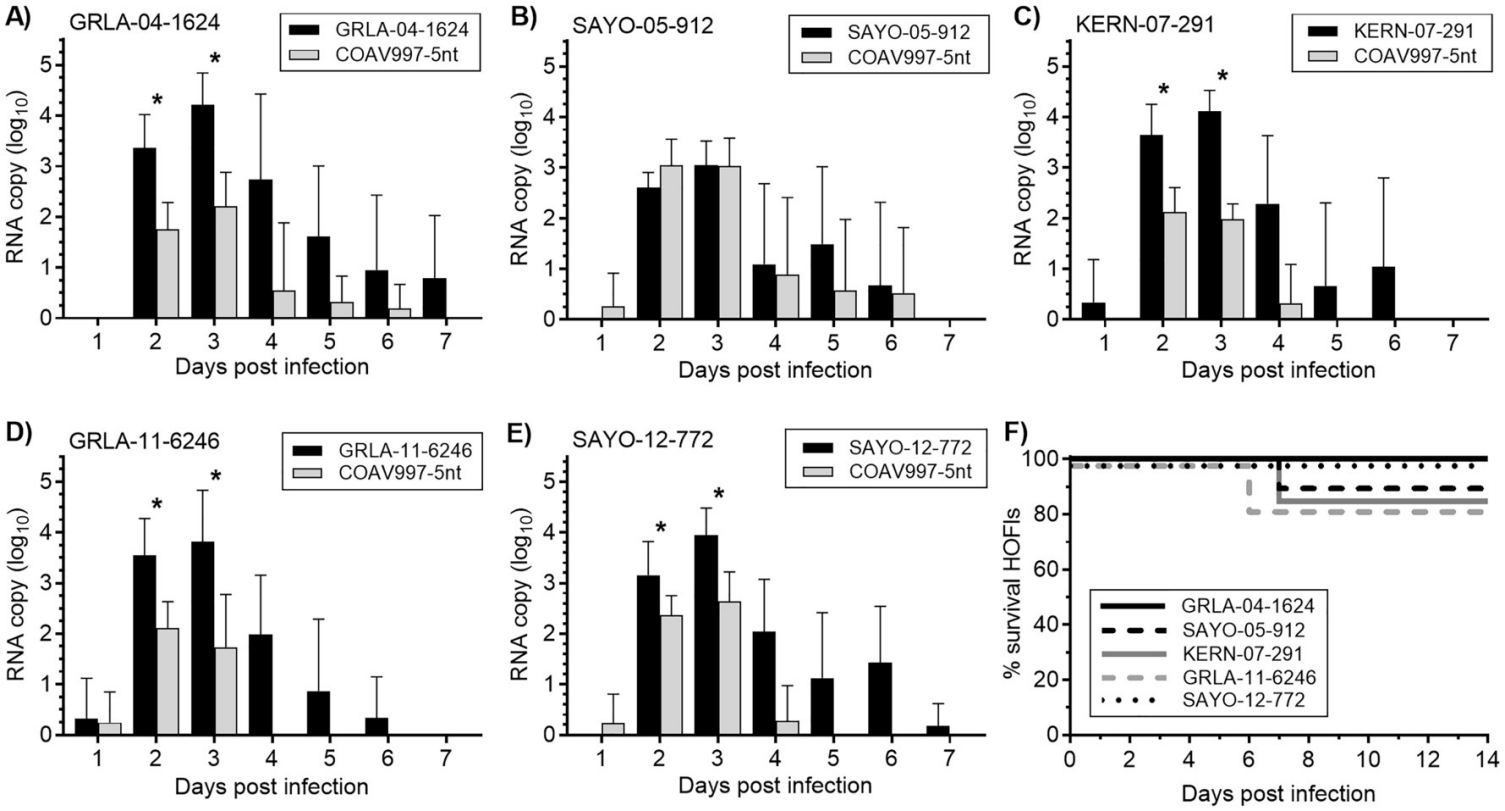
RNA profiles of dually infected HOFIs. Sera from six dually challenged HOFIs per competition group obtained between 1–7 dpi (x-axis) were analyzed by qRT-PCR for quantification of outbreak (black bars) and COAV997-5nt (grey bars) RNA. Bars with each competition group in panels A to E show the mean viral RNA copies as log_10_ in HOFI sera (y-axis) with standard deviations expessed as error bars. Degree of statistical significance between RNA copies from outbreak and COAV997-5nt virus was determined by Wilcoxon matched-pairs signed rank test and indicated for each day with an asterisk (* P ≤ 0.05); no asterisk included for P > 0.05 (no significance). Panel F shows the percent survival on 0–14 dpi.

Following serial blood collection between 1 and 7 dpi, the RNA of the outbreak strains and COAV997-5nt were quantified in HOFI sera using concurrent qRT-PCR (Fig 3). Based on RNA loads, all challenged HOFIs developed dual viremias indicating replication of both outbreak isolate and COAV997-5nt, albeit to varying levels. To determine which virus exhibited higher replicative fitness on each dpi, the mean RNA copy numbers from outbreak and COAV997-5nt viruses from HOFIs in each group were analyzed by a Wilcoxon matched-pairs signed rank test (Fig 3).

With the exception of SAYO-05-912, outbreak isolates showed higher levels of replication on 2 and 3 dpi compared to COAV997-5nt as indicated by greater mean number of RNA copies (Fig 3A and 3C–3E). In addition, the duration of viral RNA shedding was longer for GRLA-04-1624, KERN-07-291, GRLA-11-6246 and SAYO-12-772 when compared to COAV997-5nt (Fig 3A and 3C–3E) in concurrently-inoculated HOFIs. In contrast, the SAYO-05-912 outbreak isolate exhibited neutral fitness compared to COAV997-5nt between 2–6 dpi, as indicated by similar RNA copy numbers (P ≥ 0.05) and the persistence of detectable RNA from both virus strains in sera through 6 dpi (Fig 3B). Mortality following acute WNV infection was low and without significant differences among the virus competition groups (Fig 3F). There were no deaths recorded among birds in the GRLA-04-1624 and SAYO-12-772 competition groups; one HOFI each died in groups SAYO-05-912 (7 dpi), GRLA-11-6246 (6 dpi) and KERN-07-291 (7 dpi) following competition against COAV997-5nt (Fig 3F). Overt neurological signs were not observed for any of the challenged HOFIs. Across all competition groups, replication of the outbreak strains and COAV997-5nt peaked on 3 dpi (Fig 3). When comparing the means of peak replication of all outbreak isolates to COAV997-5nt, a significantly (P = 0.008) greater RNA load of 4.1±0.5 log_10_ RNA copies was observed for outbreak strains compared to 2.6±0.4 log_10_ RNA copies for COAV997-5nt (Fig 3).

**Fig 4:**
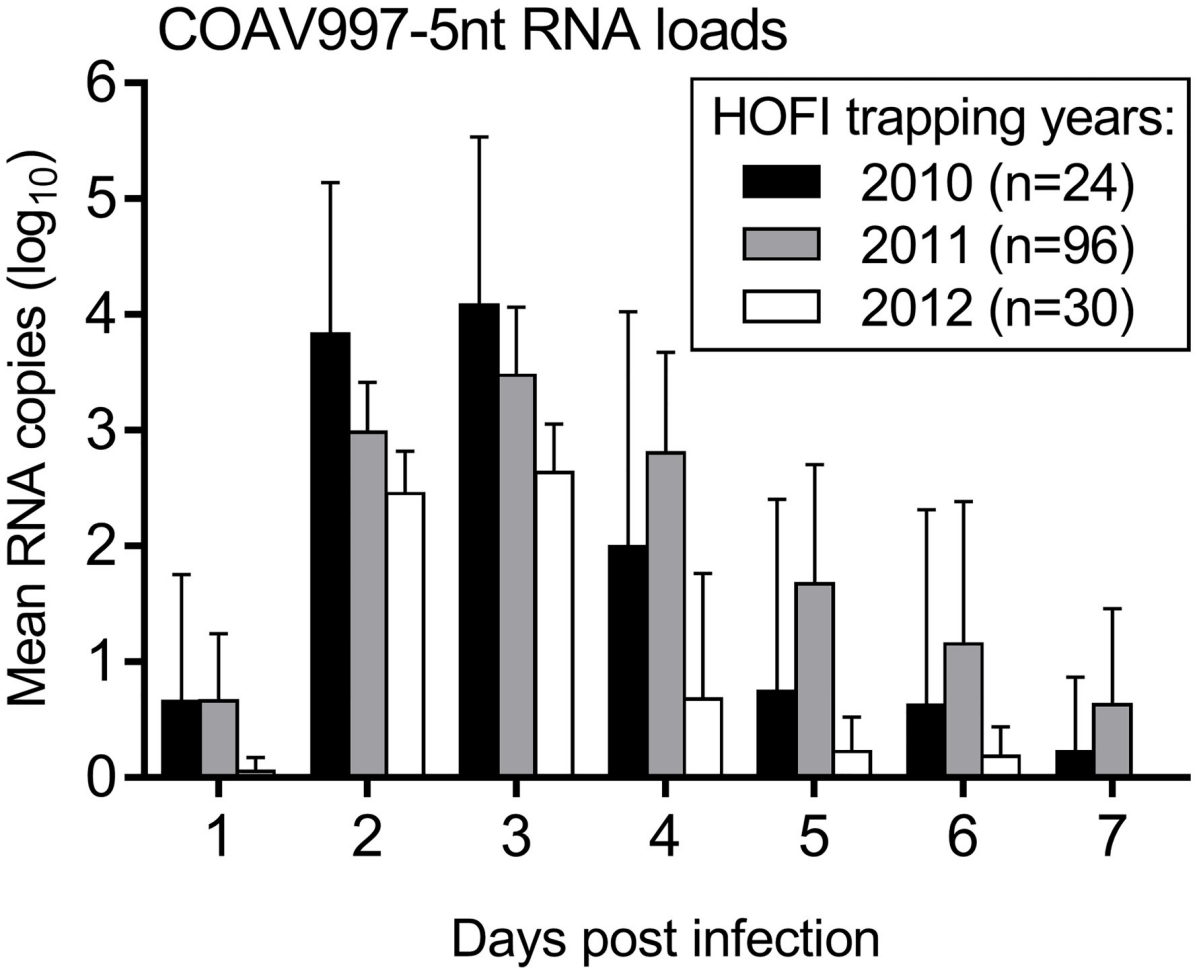
Varying competence of HOFIs to COAV997-5nt challenge. Bars show the mean daily COAV997-5nt RNA copies from hatch-year HOFIs trapped during 2010 (black color), 2011 (grey color) and 2012 (white color). The mean log_10_ RNA copies of COAV997-5nt including standard deviation (error bars) detected in HOFI sera are plotted for days 1 through 7 following competitive challenge.

Interestingly, the level of COAV997-5nt replication was lower in mixed infections during the current study (Fig 4; HOFIs 2012) when compared to similar infections in previous competitions (Fig 4; HOFIs 2010 and 2011) [21, 25] despite using the same challenge dose and COAV997-5nt stock. Because HOFIs for the three sets of competitions were trapped near Bakersfield within the same vineyards and were all hatch-year birds, the mean RNA loads from COAV997-5nt were compared with regard to year of capture (Fig 4).

Levels of COAV997-5nt replication gradually declined in hatch-year HOFIs captured between 2010–12, particularly when comparing peak RNA loads on 3 dpi by Mann-Whitney test. HOFIs from 2012 developed peak COAV997-5nt RNA loads of 2.6±0.4 log_10_, whereas HOFIs from 2011 showed on average significantly higher RNA loads of 3.8±0.6 (P = 0.004). In contrast, HOFIs from 2010 were more competent developing significantly higher peak COAV997-5nt RNA loads of 4.1±1.5 compared to 2011 (P = 0.01) and 2012 in particular (P = 0.009) (Fig 4).

## Discussion

The goal of the current study was to evaluate the replicative fitness of WNV isolated during five California outbreaks from 2004 to 2012 by competition against COAV997-5nt in *Cx*. *tarsalis* and HOFIs. Our findings demonstrated that isolates from four out of five outbreaks replicated more efficiently in HOFIs compared to COAV997-5nt (Fig 3), but mortality and viral RNA loads were significantly lower compared to previous WNV isolates [21], presumably due to decreased competence of HOFIs trapped during 2012 (Fig 4). Similar to our previous study [23], competition in *Cx*. *tarsalis* resulted in varying numbers of singly and dually infected mosquito bodies, but infection and expectoration rates were not dominated by one virus strain over the other (Figs 1 and 2). Similar to our previous findings [21], the amount of viral RNA found in *Cx*. *tarsalis* bodies was not a reliable determinant of viral fitness, because RNA copy differences between outbreak and COAV997-5nt viruses were either not significant or not proportionate to the virus that infected greater numbers of bodies and expectorants (Figs 1 and 2). Salivary glands are the final site of WNV replication within *Cx*. *tarsalis* and secretion of infectious virus in the expectorant determines potential WNV transmission during subsequent blood feeding on susceptible hosts [28, 29]. Although significantly greater COAV997-5nt than KERN-07-291 RNA copy numbers were found in mosquito bodies (Fig 2C), equal numbers of females expectorated either KERN-07-291 or COAV997-5nt viruses (Fig 1B), indicating equal transmission, despite greater COAV997-5nt replication. It is possible that the higher infection rate and subsequently larger sample size may have resulted in this difference compared to other groups. Overall, competitions in *Cx*. *tarsalis* did not result in the complete displacement of the outcompeted virus as both strains were retained and transmitted within all mosquito groups (Fig 1).

Mortality in HOFIs during the current five outbreak competitions was not different from mortality rates observed with COAV997 and COAV997-5nt infections alone and combined [25], and was decreased compared to several enzootic California isolates evaluated from 2007–08 [21] and to the WNV NY99 strain in HOFIs collected during 2003 [30]. Although virulence and mortality rates were not elevated in HOFIs in the current competitions, WNV outbreak isolates (except for SAYO-05-912) replicated to higher RNA copy numbers compared to COAV997-5nt. The magnitude and duration of RNA present in sera potentially indicated a commensurate viremia which would be important for oral mosquito infection and subsequent transmission [10]. Interestingly, peak RNA loads were significantly lower for both COAV997-5nt and outbreak isolates in HOFIs that were trapped in 2012 compared to RNA loads in HOFIs collected from the same sites in 2010–11 when co-infected with the same stock of COAV997-5nt, wild type WNV of similar fitness, and using the same challenge protocol [21]. This may be due to decreased competence of the local HOFI population as a consequence of rising natural resistance against WNV and/or a result of host-pathogen co-evolution previously observed in HOSPs [15]. Following invasion of the NY99 genotype into North America in 1999, high avian virulence and mortality were hallmarks of WNV infection, with HOFIs developing viremias up to 8 log_10_ PFU per mL sera [22, 31]. With the emergence of the WN02 genotype; however, the host competence indices of HOSPs for NY99 significantly decreased over time, with lower viremia and mortality rates compared to infection with more recent SW03 and WN02 genotype isolates [15]. Between 2003 and 2012, there was a 22% WNV infection prevalence in dead California HOFIs [11] and a decline in population abundance since the arrival of WNV in the state [31], possibly increasing selection pressure on these birds for resistance. This could be one reason for observing reduced HOFI competence for WNV in the current study. Our competition model has the advantage of revealing fitness differences among WNV strains through intra-host competition, therefore minimizing the confounding effects of differences in host immune response. This also presented a limitation of our study, because we used a single moderately competent avian host and vector species in our *in vivo* competition model without inclusion of low- or high-competence hosts and vectors.

Collectively, the competitive fitness of outbreak WNV isolates associated with five California epidemics between 2004 and 2012 was not markedly different compared to enzootic wild type isolates [21] indicating that additional ecological and environmental factors were necessary to trigger WNV amplification to epidemic levels. Similar to the 2012 WNV epidemic in the Dallas-Fort Worth area which was not tied to increased WNV virulence[32], above-average temperatures and reduced rainfall may have enhanced transmission by mosquitoes and warrants further study.
